# Depression: an exploratory parallel-group randomised controlled trial of Antenatal guided self help for WomeN (DAWN): study protocol for a randomised controlled trial

**DOI:** 10.1186/s13063-016-1632-6

**Published:** 2016-10-18

**Authors:** Kylee Trevillion, Jill Domoney, Andrew Pickles, Debra Bick, Sarah Byford, Margaret Heslin, Jeannette Milgrom, Rachel Mycroft, Carmine Pariante, Elizabeth Ryan, Myra Hunter, Louise Michele Howard

**Affiliations:** 1 Institute of Psychiatry, Psychology and Neuroscience, King’s College London, London, UK; 2 Florence Nightingale Faculty of Nursing and Midwifery, Division of Women’s Health, King’s College London, London, UK; 3 University of Melbourne and Parent-Infant Research Institute, Melbourne, Australia

**Keywords:** Depression, Pregnancy, Guided Self-Help, Randomised controlled trial

## Abstract

**Background:**

Depression is a common antenatal mental disorder and is associated with an increased risk of adverse effects on the fetus and significant morbidity for the mother; if untreated it can also continue into the post-natal period and affect mother-infant interactions. There has been little research evaluating the effectiveness or cost-effectiveness of antenatal psychological interventions for antenatal depression, particularly for mild to moderate disorders. International guidelines recommend a stepped care approach starting with Guided Self Help, and the aim of this exploratory trial is to investigate Guided Self Help modified for pregnancy.

**Methods:**

The DAWN trial is an exploratory randomised controlled trial of the effectiveness and cost-effectiveness of antenatal Guided Self Help, modified for pregnancy and delivered by National Health Service Psychological Wellbeing Practitioners. Antenatal Guided Self Help, in addition to usual care, is compared with usual care for pregnant women diagnosed with mild to moderate depression and mixed anxiety and depression, using the Structured Clinical Interview for DSM-IV Disorders. Modifications for pregnancy include perinatal mental health training, addressing pregnancy-specific worries and including sections on health issues in pregnancy and planning for parenthood. Women allocated to Guided Self Help will be seen for up to eight sessions by a Psychological Wellbeing Practitioner (including an initial assessment session); there will also be an appointment at 12 weeks after delivery. Research measures including the Edinburgh Postnatal Depression Scale (primary outcome) and other measures of depression, anxiety, quality of life and service use will be collected from women before random allocation, 14 weeks after random allocation and at 12 weeks after delivery. Potential psychological mechanisms of the intervention will be explored using the Pregnancy-Related Thoughts Questionnaire and the Metacognitive Awareness Questionnaire.

**Discussion:**

The DAWN trial is the first exploratory trial to investigate the efficacy of antenatal Guided Self Help for pregnant women with mild to moderate depression meeting DSM-IV diagnostic criteria. Recruitment started January 2015 and is expected to be completed by July 2016.

**Trial registration:**

ISRCTN registry: ISRCTN83768230. Registered on 8 August 2014.

**Electronic supplementary material:**

The online version of this article (doi:10.1186/s13063-016-1632-6) contains supplementary material, which is available to authorized users.

## Background

Pregnancy does not appear to be protective against the persistence or development of psychiatric disorders [1]. Depression is the most common antenatal mental disorder with a 9 % period prevalence [1], which is similar to that for non-pregnant childbearing-aged women. Depression in pregnancy can have a considerable impact upon the woman and her family, and is associated with an increased risk of (1) adverse effects on the fetus, including low birth weight and preterm delivery [2]; (2) infant deaths [3]; (3) post-natal maternal psychopathology [4–6]; (4) subsequent behavioural/emotional problems in the child and adolescent [7]; and (5) negative impacts on other family members [8]. Such adverse outcomes are not inevitable, and early effective interventions could mitigate these effects. Since mental disorders in pregnancy are also associated with other risk factors that may lead to adverse outcomes, particularly smoking [9, 10], socio-economic deprivation [11], obesity [12] and domestic violence [13, 14], consideration of associated risk factors should also be included in the development of interventions. International guidelines recommend that there should be a higher threshold for medication than at other times in a woman’s life due to the potential risk to the fetus, as well as a lower threshold for offering psychological therapies [15]. To date there has been little research evaluating the effectiveness of antenatal psychological interventions for the most common disorder: mild and moderate antenatal depression.

Cochrane reviews have reported limited evidence on the effectiveness of small trials of interpersonal therapy, massage and acupuncture [16, 17], and there have only been three recently completed pilot randomised controlled trials of cognitive behavioural therapy (CBT) [18–20]. To our knowledge, there has been no evaluation of less intensive psychological interventions for women diagnosed with mild and moderate depression in the antenatal setting — interventions which may be more cost-effective.

The National Institute for Health and Care Excellence (NICE) [15] recommends a stepped care approach for depression, starting with Guided Self Help, a low-cost intervention for mild and moderate depression which is delivered by Psychological Wellbeing Practitioners. Recent UK national policy highlights the importance of improving access to psychological therapies for women in the perinatal period [21]. A recent systematic review and meta-analysis of Guided Self Help for mild and moderate depression found evidence of effectiveness post-treatment but limited effectiveness at long-term follow-up, with variability across different settings [22]; no trials in perinatal settings were included. However, an Australian study of an antenatal supported self help intervention for the prevention of perinatal common mental symptoms reported reduced depressive symptoms at 12 weeks post-delivery [23]. Moreover, in the antenatal period even short-term improvement in mild disorders is important, as there is evidence that mild disorders can adversely affect fetal outcomes [2]. Although psychological interventions in the antenatal setting which are less intensive may be more cost-effective for mild and moderate antenatal depression, there has been no evaluation of this approach. We therefore propose to evaluate a new form of Guided Self Help for antenatal depression in women using UK National Health Service (NHS) maternity services in an exploratory randomised controlled trial (RCT).

The objectives of this exploratory trial are to:

1. Establish recruitment and follow-up rates
2. Indicate levels of compliance with the intervention
3. Indicate for whom the intervention is most suitable
4. Provide preliminary evidence for the efficacy of the intervention (estimating treatment effect sizes)
5. Provide preliminary evidence for the cost-effectiveness of the intervention compared to usual care
6. Explore the psychological mechanisms of the intervention.

We will test our procedures within large maternity services in English NHS settings [24]. The NHS is the publicly funded healthcare system for England. It provides healthcare to every legal resident in the United Kingdom, with mental health and primary care among the services free at the point of use.

There are four specific main aims of this trial: (1) to establish that the trial procedures work (and fine-tune where necessary) so that a Phase III trial can follow; (2) to evaluate whether antenatal Guided Self Help is beneficial in improving depressive symptoms for women with antenatal depression; (3) to evaluate whether antenatal Guided Self Help has the added benefit of improving other outcomes, including post-treatment and post-delivery psychological symptoms, post-delivery bonding and quality of life; and (4) to explore whether antenatal Guided Self Help is likely to be cost-effective compared to usual care. The latter two aims will also estimate treatment effect sizes.

The following hypothesis will be tested:

Women with mild or moderate antenatal depression treated with Guided Self Help will have significantly lower Edinburgh Postnatal Depression Scale (EPDS) depressive symptoms at 14 weeks post-randomisation compared to women with mild or moderate antenatal depression receiving usual care.

## Methods/design

This study has been designed according to the Standard Protocol Items: Recommendations for Interventional Trials (SPIRIT) statement. Additional file 1: Table S1 presents the SPIRIT checklist, and Additional file 2: Figure S1 presents the SPIRIT figure for the schedule of enrolment, intervention and assessments.

The trial is a multi-centre Phase II exploratory randomised controlled trial with two parallel groups and a primary endpoint of EPDS depressive symptoms at 14 weeks post-randomisation.

Pregnant women with depression will be randomly allocated to either Guided Self Help plus usual care or to usual care alone (i.e. treatment as usual). Randomisation, stratified by type of depression (i.e. mild or moderate depression, or mixed anxiety and depression), will be performed using block randomisation of varying sizes with a 1:1 allocation.

### Study setting

The study setting is inner city English NHS maternity services in South East London serving an ethnically and socially diverse population.

### Eligibility criteria

#### Inclusion criteria

Women must have the following characteristics prior to randomisation:

1. Aged ≥16 years
2. Pregnant, not exceeding 26 weeks gestation
3. Meet criteria for Diagnostic and Statistical Manual of Mental Disorders (DSM)-IV depression (mild or moderate major depressive disorder, or mixed anxiety and depressive disorder) on the Structured Clinical Interview DSM-IV (SCID)
4. Able to provide informed consent.

#### Exclusion criteria

The exclusion criteria are as follows:

1. Pregnant women receiving cognitive behavioural therapy or another individual or group psychological therapy
2. Pregnant women taking antidepressants
3. Pregnant women suffering from psychosis, current eating disorder, borderline personality disorder or current post-traumatic stress disorder, or receiving care from secondary mental health services
4. Pregnant women who are unable to complete questionnaires or follow the trial workbook in English
5. Pregnant women who endorse ‘yes, quite often’ on question ten of the Edinburgh Postnatal Depression Scale (EPDS) or other measures of suicidality (e.g. at least two items of the SCID on suicidality) [25].

### Recruitment

Recruitment started in January 2015 and is expected to be completed by July 2016. The target sample size is 110 women. At the point of submission, a total of 50 women were recruited to the trial.

Women will be recruited to the trial in one of three ways:

1. Women attending an antenatal booking clinic in a South East London maternity service who complete the Whooley depression screening questions used routinely in UK maternity services [26] will be approached by a research midwife and asked to take part in a related study on wellbeing in pregnancy, which includes measurement of the prevalence of depression (REC reference: 14/LO/0075). Women who consent to take part in the wellbeing in pregnancy study and who are identified as having depression on the SCID will be asked by a research midwife if they would like to take part in this trial.
2. A woman may be referred to the trial by a midwife in South East London maternity services at a later point in pregnancy (up to 26 weeks gestation) if the midwife suspects that the woman is depressed and may be suitable for the trial. Women can also self-refer (as happens in routine Improving Access to Psychological Therapies (IAPT) care); advertisements about the study will be placed in antenatal clinics, scanning departments and other settings for local pregnant women, e.g. children’s centres. The research midwife will administer the baseline measures to assess eligibility of women for the trial.
3. A woman may self-refer to the trial should she feel she is experiencing symptoms of depression. An advertisement for self-referral to the trial will be positioned in maternity waiting rooms and women’s booking packs, and the research midwife will administer the baseline measures to assess eligibility of women for the trial.

### Assessment of eligibility

The research midwife will outline to women the trial processes and procedures as described in the participant information sheet, and answer any questions that arise. Women will have a minimum of 24 hours to consider if they wish to participate in the trial.

### Randomisation

Women will be randomly assigned to: (1) Guided Self Help (delivered by a Psychological Wellbeing Practitioner) plus usual care or (2) usual care alone (i.e. treatment as usual). Randomisation will be conducted independently of the trial investigators via the UK Clinical Research Collaboration (UKCRC) registered King’s Clinical Trials Unit bespoke web-based central randomisation service. Using a sampling fraction and cut-offs determined from the SCID instrument, collected during a related study on wellbeing in pregnancy (REC reference: 14/LO/0075), we will recruit women in three strata of mild or moderate depression or mixed anxiety depression. To ensure balance, the web-based central randomisation service will allocate women to trial groups (with a 1:1 allocation) using randomly varying blocks, stratified by severity of depression (assessed using scores on the SCID depression scale). The block sizes will not be disclosed, to ensure concealment.

### Allocation to trial groups

Once an eligible woman has given her consent and completed baseline measures, she will be randomised to either Guided Self Help plus usual care, or usual care alone. The research midwife will then communicate information about the allocation group to the participant and, for women allocated to Guided Self Help, will arrange an appointment with the Psychological Wellbeing Practitioner (PWP) to start the intervention.

This trial, being embedded within a related population screening study on wellbeing in pregnancy (REC reference: 14/LO/0075), will allow us to compare the baseline and outcomes of those in the treatment-as-usual group and those receiving treatment as usual though not participating in the trial.

See Additional file 3: Figure S2 for a protocol flow diagram.

## Intervention

### Guided Self Help interventions for depression

Guided Self Help is delivered within the Improving Access to Psychological Therapies (IAPT) programme, which is a large-scale initiative that aims to increase the availability of NICE-recommended psychological treatments for depression and anxiety disorders within NHS-commissioned services in England. It was originally launched in 2008, and key successes in the first three full financial years include more than 1 million people entering treatment, 680,000 people completing treatment and recovery rates in excess of 45 % (with around 65 % showing significant improvement) [24]. IAPT is continuing to expand with plans to be available to at least 15 % of the adult population and to deliver recovery rates of 50 % or more by March 2015 [24].

Two half-time NHS PWPs — Agenda for Change band five, with a post-graduate diploma in low intensity interventions — will be seconded from the IAPT programme and will deliver the trial intervention of Guided Self Help modified for pregnancy. PWPs are UK NHS health service employees who deliver evidence-based low intensity cognitive behavioural therapy (CBT) interventions within the IAPT programme. They have experience in delivering treatment based on Guided Self Help manuals, and in supporting patients’ use of Guided Self Help manuals and relevant freely available resources through regular contact. PWPs are used to carrying a high volume caseload of patients requiring low intensity interventions, and they are experienced in monitoring progress and assessing changes in symptoms; they may signpost to other services if necessary. As part of their role within the IAPT programme, PWPs are required to collect a number of standardised measures including the Patient Health Questionnaire (PHQ-9), Generalised Anxiety Disorder (GAD-7), the Work and Social Adjustment Scale and Phobia Scales. The PWPs are required to possess a range of perinatal competencies in order to successfully deliver the Guided Self Help components of the trial (see the list of competencies in the Appendix).

### Modified Guided Self Help (with usual care) for antenatal depression

A workbook appropriate for pregnancy (available from the corresponding author on request) was developed. In order to establish the components of the intervention, a literature search was carried out to identify studies on risk factors for antenatal depression, moderating factors for poor child outcomes and qualitative studies on women’s experience of antenatal depression. Following this process, key components identified included:

1. Psycho-education about the symptoms and causes of depression with a focus on factors related to pregnancy
2. An introduction to the cognitive behavioural model and the links between thoughts, feelings and behaviours, and cognitive-behavioural strategies for managing mood, including activity planning, problem solving and thought challenging
3. Managing relationships in the perinatal period, including increasing social support,
4. the development of maternal-fetal attachment and reflecting on how we learn to be parents
5. Health and lifestyle factors, including issues involving smoking and nutrition
6. Preparing for parenthood.

The workbook was divided into six chapters, each including a mixture of educational material, testimonials from pregnant women and exercises to be completed by participants. Exercises were based on cognitive behavioural skills, starting with more behavioural interventions such as activity planning and problem solving, and moving on to more cognitive skills such as thought challenging and identifying underlying rules for living. At the end of each chapter a list of homework tasks were suggested, and throughout the workbook there were links to other sources of support, e.g. websites, organisations which provide support or advice to pregnant women, children’s centres and antenatal classes.

Recent evidence suggests that a minimum of four sessions is necessary to achieve good rates of change for low intensity interventions and that dose-response reduces after six sessions [27]. The workbook was therefore designed to be delivered across six sessions; adding an introductory session and a final maintenance session led to an eight-session intervention. Current IAPT practice is to offer sessions lasting between 30–45 minutes, either on the telephone or face to face.

During development, drafts of the workbook were sent to an advisory panel of perinatal experts (psychologists, psychiatrists and midwives) for comments and feedback. A draft was also sent to a panel of service users who were part of an advisory group to a larger perinatal study. These women provided feedback on wording, relevance and use of examples and layout, and the workbook was modified accordingly. The workbook was piloted by one of the authors (JD) in routine IAPT care; ten women took part in the pilot to assess the feasibility of delivering the intervention during pregnancy, the acceptability of the content to pregnant women and preliminary outcomes. Based on women’s feedback, a number of amendments were made: the text was edited to reduce the number of words; the title was changed to ‘Wellbeing in Pregnancy’ (several women commented on the original title of the workbook, ‘Antenatal Depression’, which they felt was unhelpful due to possible stigma and their associations with more severe forms of perinatal disorders); specific references were made to how different sections were relevant to women who already have children; and the health chapter was adapted to be more focussed on overcoming barriers to good health behaviours (several women felt the chapter on health repeated messages they had from midwives and had less of a warm tone than other chapters). Women reported needing the sessions to motivate them and engage with the material; i.e. they indicated that they may not have completed the workbook on their own. The routinely collected scores on measures of anxiety (GAD-7) and depression (PHQ-9 and EPDS) reduced following the intervention.

Prior to delivering this Guided Self Help intervention within this pilot RCT, the two PWPs were trained in the modified intervention (and subsequently supervised on a weekly basis) by an experienced perinatal clinical psychologist. They were also trained in associated areas relevant to perinatal clinical work including safeguarding, violence and abuse, smoking cessation and the mother-child relationship [21]. The two PWPs will collect the same standardised measures as they would within an IAPT setting — Patient Health Questionnaire (PHQ-9), the Generalised Anxiety Disorder scale (GAD-7), the Work and Social Adjustment Scale and Phobia Scales — at every Guided Self Help session they have with trial participants.

The Guided Self Help (with usual care) intervention for antenatal depression comprises an initial session at the beginning of therapy followed by up to eight 30-minute sessions, delivered weekly where possible. The PWPs will also conduct an additional session, at six to eight weeks post-delivery (i.e. before the post-delivery research outcomes are collected). The first Guided Self Help session will be delivered face to face, and the subsequent sessions will either be delivered face to face or over the telephone, based on the participants’ preferences.

Based on existing evidence from trials of Guided Self Help [27], a minimum number of four sessions is required to determine reliable and clinically significant improvements. Women who show no improvement in symptoms at the end of the Guided Self Help sessions and who want further support will be referred by the PWP either for high intensity interventions or to their general practitioner (GP), as is the usual clinical practice. Women who show significant deterioration in symptoms on the routinely collected measures — at any point during or at the end of the Guided Self Help sessions — will be discussed in the PWPs’ clinical supervision (or in more urgent situations, the nominated senior psychologist available for emergencies) and referred as appropriate to GPs, high intensity psychologists or secondary care.

### Treatment as usual

The findings of local audits and a recent study [18] indicate that very few pregnant women are referred for psychological therapy in routine practice. We will record any referrals that occur, treatment received and other interventions accessed (e.g. third sector support, antidepressants) for women in both groups of the trial (at the two follow-up points).

## Outcomes

### Primary outcome

The primary outcome is the score for Edinburgh Postnatal Depression Scale (EPDS) depressive symptoms at 14 weeks post-randomisation.

### Key secondary outcomes

#### At 14 weeks post-randomisation

At 14 weeks post-randomisation, the secondary outcomes are:

1. Proportion of participants meeting PHQ-9 criteria for depression (i.e. score of ≥10)
2. Proportion of participants meeting GAD-7 criteria for anxiety (i.e. score of ≥8).

#### At 3 months post-delivery

At 3 months post-delivery, the secondary outcomes are:

1. EPDS score
2. Parenting stress.

See Additional file 4: Table S2 for further details of the measures and times of assessment.

## Measures

Data will be collected at the following time points: baseline, 14 weeks post-randomisation and 12 weeks post-delivery (see Additional file 4: Table S2 for a full list of the study measures). Baseline measures will be collected by researchers prior to the assignment of participants to the trial groups. Baseline interviews may take up to 45 minutes if women have been referred to the trial by clinic midwives or via self-referral; these women will need to undergo a detailed assessment of their eligibility for the trial. For women taking part in the related study, the interview will take between 5 and 8 minutes, as they will have already completed a detailed assessment of their eligibility for the trial and will just need to be asked some additional measures. The 14-weeks post-randomisation interview may take up to 30 minutes and the 3-months post-delivery interview up to 40 minutes. All follow-up measures will be collected by another researcher who will be blind to the allocation status of the participant. The researcher will ask the participant not to mention to which group she was allocated. A test of the success of masking will be carried out; at the end of each follow-up interview, the researcher will document his/her views on which arm of the trial he/she believes participants have been allocated to. At the end of the trial, we will compare the actual allocation status of participants against the researcher’s estimated allocation status to determine the strength of blinding in the trial.

The following data will be extracted from maternity records:

1. Mode of delivery; maternal ethnicity; parity and pre-pregnancy body mass index (estimated at booking); fetal growth data as available from routine scans
2. Infant outcomes: Apgar scores; birthweight; sex; gestational age to calculate customised birthweight centiles adjusted for maternal age; need for resuscitation; days in neonatal unit; growth weight and height centiles over first 12 weeks where available from routine measures in primary care.

The PWPs will also collect data using the PHQ-9 [28], GAD-7 [29], IAPT Phobia Scale [30] and IAPT Work and Social Adjustment Scale [31] at each session delivered to women in the Guided Self Help plus usual care group, as this is normal practice in all IAPT services.

### Process evaluation

A fidelity rating scale has been developed to rate adherence to the Guided Self Help components by the PWPs. We aim to audio-record five initial sessions for each PWP and rate their adherence to the intervention components using a checklist corresponding to the content of the workbook modules. Rating of fidelity will be carried out by two independent psychologists. Further ratings on a random sample of 20 sessions will ensure quality control and fidelity. The scale was designed to assess adherence to the key components of the intervention. It includes items such as setting an agenda/orienting the client to the session, discussing questionnaire scores, covering material from the workbook and reviewing and setting homework. It was necessary to use a slightly adapted version of the scale for the first and last sessions because of intrinsic differences (e.g. there was no homework to review from a previous session in the first session). Each item is rated according to its presence or absence from the session, with the item being scored if there was any evidence that it had been included. A rating of 80 % adherence indicates high fidelity.

Additionally, it was thought important to have a measure of the ability of the PWPs to develop a positive therapeutic alliance. Research suggests that the relationship between client and therapist is a key variable in relation to therapy outcomes ([32, 33], but see also [34]). The Cognitive Therapy Scale Revised (CTS-R) [35] is a widely used and well-validated instrument for evaluating therapist competency and adherence to CBT principles. It was not appropriate to use a full CTS-R, because the intervention was not full cognitive behaviour therapy but rather a cognitive behaviourally based Guided Self Help package. Item 5 of the scale concerns ’interpersonal effectiveness’ defined as ’the ability of the therapist to form a good relationship with the patient’. This includes the ability to put the client at ease, to facilitate disclosure of difficult material and to exhibit the nonspecific factors of empathy, genuineness and warmth delineated by Carl Rogers [36]. It was decided to use this item of the scale, rated from 0 to 6, as an indication of the extent to which PWPs were able to respond to their clients and build a positive therapeutic alliance. Scores on the scale should approximate to a normal distribution with a mid-point of 3, with very high scores reserved for expert competency, particularly in the face of difficulties. When the full scale is used, a mean score of 3 on each item is taken to indicate competency. Because the full scale could not be used, the ratings provide an indication of interpersonal effectiveness but do not represent the broader picture of CBT competencies assessed by the full scale. The PWP will also collect data on the mode of delivery of Guided Self Help sessions (i.e. face to face or via telephone).

Levels of compliance will be assessed using data collected by the PWPs on the number and length of Guided Self Help sessions offered and the number of sessions attended/not attended or cancelled.

Descriptions of treatment as usual will be recorded, including the frequency of contact with health/social care services (using the Adult Service Use Scale (AD-SUS)) at 14 weeks post-randomisation and 12 weeks post-delivery.

Qualitative data will be collected from interviews with a purposive sample (with respect to ethnicity, age and parity) of 20 women, after completion of the 3-months post-delivery research interviews. A brief topic guide will be used to ask participants for feedback about their expectations and experiences of participating in the intervention (and/or other care) and using maternity and primary care services.

## Sample size

This is primarily a feasibility trial, but we also carried out a sample size calculation to allow us to calculate what preliminary evidence we could generate on efficacy. Assuming a correlation of 0.4 between baseline and outcome EPDS symptom score, a two-arm parallel-group design with 52 women in each arm, the Stata procedure *sampsi* gives 79 % power to detect a difference of 0.5 SD using ANCOVA and a two-tailed test using a 95 % significance level (based on an effect size of 0.31, as this was found in a systematic review of Guided Self Help in non-maternity settings [22]). We aim to recruit 110 women. The study would have 66 % power to detect a difference in the caseness rate of recovery of 35 % in controls versus 60 % in the Guided Self Help arm.

## Statistical analysis

Analysis will be by intention to treat; missing data will be imputed. The planned strategy for handling missing data at the item and scales will depend on the amount of missing data observed and the planned analyses for the outcomes. To ensure the same strategy is followed across all scales reported in the principle paper(s), any guidance given by authors of validated questionnaires will supersede the methods outlined herein.

If any of the baseline measures can be hypothesised to predict missing scale outcomes, it would be advantageous to include them in the modelling. To allow for this, any baseline measure considered as a covariate in the main modelling must be imputed to a full dataset. Missing baseline covariate item data will be imputed using mean imputation per participant (pro rating) or using the *ice* command in Stata to make a single imputation. There will be missing data in post-treatment outcome variables as participants are lost to follow-up. The regression analyses are based on maximum likelihood, and resulting inferences are valid provided the missing data generating mechanism is missing at random (MAR); that is, missingness is predicted only by variables that are included in the model, including earlier values of the outcome variable. We will empirically assess using logistic regression whether any baseline variables predict missingness, and should this be the case, we would condition on such variables by including them in the statistical model. Where post-randomisation variables predict missingness, we will make use of the MAR properties of (restricted) maximum likelihood random effects estimators for repeated measures.

Although the main aim of the trial is to assess feasibility, an exploratory primary outcome analysis will be carried out using a regression of the EPDS depression symptom score using baseline symptom score as a covariate (ANCOVA), with confidence intervals (CIs) calculated using bootstrap. Because the trial is embedded within a screening study, we will estimate the effect of the treatment on diagnostic rates of depression in the pregnant population using multiple imputation from EPDS symptom scores and screening scores available in the whole screened population.

An odds ratio and 95 % CI for recovery, defined within IAPT as a PHQ-9 score of ≥10 at 14 weeks post-randomisation, will be estimated. We will compare groups for continuous scores on the EPDS at 14 weeks post-randomisation and 3 months post-delivery. The variance estimates will assist in power calculations for a definitive trial. Supplementary analyses will estimate complier average causal effects for a binary measure of compliance to treatment using the instrumental variable method. Therapist effects will be evaluated. Infant growth will be explored using random effects growth curve models (*xtmixed* in Stata) to compare birth and growth velocities of the groups.

Qualitative data will be coded in NVivo8 [37] following the principles of framework analysis [38], which includes the identification of a thematic framework, the abstraction and synthesis of key themes and the mapping and interpretation of the dataset as a whole. Qualitative results will be discussed within the context of quantitative data interpretation.

## Economic evaluation

The economic evaluation will take the NHS and personal social services perspective preferred by NICE [39]. Resource use data from the AD-SUS (see Additional file 4: Table S2 for full details of the AD-SUS measure) will be combined with unit costs to calculate total costs of the Guided Self Help and usual care groups. The cost of Guided Self Help will be calculated using a micro-costing approach based on the PWPs’ salary, including relevant on-costs and overheads. Unit costs of other services will be taken from national published sources.

Exploratory economic analyses, on an intention-to-treat basis, will include tests for differences in cost using standard parametric *t* tests with the validity of results confirmed using bias-corrected, nonparametric bootstrapping (repeat re-sampling) [40]. Cost-effectiveness will be assessed through the calculation of incremental cost-effectiveness ratios [41] and will be explored in terms of quality-adjusted life years using the SF-6D (derived from the 36 item Short Form Health Survey (SF-36)). A sensitivity analysis will be performed using the EuroQoL five dimensions questionnaire (EQ-5D), and a formal assessment of the validity and responsiveness of both the SF-6D and EQ-5D will be undertaken following standard methods [42, 43]. All regressions to calculate mean differences in costs will be repeated with the further inclusion of covariates for baseline clinical measures. Uncertainty around the cost and effectiveness estimates will be represented by cost-effectiveness acceptability curves [44]. Should the results of the trial support the use of Guided Self Help as an effective and cost-effective treatment option for mild and moderate antenatal depression, data from the trial will be used to re-run the economic decision model from a related study (WENDY- http://www.kcl.ac.uk/ioppn/depts/hspr/research/CEPH/wmh/projects/A-Z/WENDY-well-being-in-pregnancy-in-an-inner-city-maternity-service.aspx) in order to explore the relative cost-effectiveness of the alternative screening approaches when Guided Self Help is incorporated into the treatment pathway for this group of women.

## Discussion

The DAWN trial is the first exploratory trial to our knowledge to investigate the efficacy of antenatal Guided Self Help for pregnant women diagnosed with mild to moderate depression. As part of a larger National Institute for Health Research (NIHR)-funded programme of research (see http://www.kcl.ac.uk/ioppn/depts/hspr/research/CEPH/wmh/projects/A-Z/esmi.aspx), independent oversight is provided by a Programme Steering Committee, Programme Data Monitoring and Ethics Committee (DMEC) and Trial Steering Committee (TSC). The UKCRC registered King’s College London (KCL) Clinical Trials Unit provides web-based data entry systems using the InferMed MACRO data entry system which is compliant with Good Clinical Practice (GCP) and Food and Drug Administration (FDA) 21 Code of Federal Regulations (CFR) Part 11. No interim analyses are planned. Only the trial investigators and the TSC and DMEC, if any adverse or other unintended effects are identified, will have access to participants’ personal data. We will collect, assess, report and manage any adverse events that may occur. Any adverse events or other unintended effects of the trial intervention or conduct will be recorded and reported to the DMEC (DMEC charter available from author).

## Ethical issues

A number of practical measures have been taken to minimise distress and risk to participants. These include the following:

1. When taking consent, researchers will explain to participants that they can take time in answering questions and do not have to answer questions that they do not want to.
2. At the baseline interview, researchers will ask participants if they would like to nominate a contact for support, should they become distressed.
3. During the interview, researchers will closely monitor participants for signs of distress and, if observed, will take appropriate action, including asking the participant if she would like to take a short break, skipping questions that cause particular distress, or offering to complete the interview at another time.

Safety protocols have been developed to ensure that participants, their families and the researcher remain safe when making contact, conducting research and afterwards. On initial contact, researchers will establish an appropriate contact number and time for future contact between themselves and participants; if participants would like to keep their participation in the research confidential from other people, researchers will establish how to manage the situation if conversations are overheard (e.g. they can start discussing safety of electrical appliances in the home). This is done because there is a risk that telephone conversations may still be overheard. At all points of contact, researchers will confirm that they are speaking with the participant and will not leave information with any other household member, unless the participant has specified that this is okay. The identity of the researchers will not be given to any caller until the identity of the caller has been established. Researchers will ensure that the location(s) where an interview takes place is private and secure and cannot be overheard. Interviews will be conducted either in a secure room at the NHS Trust or, if deemed safe, at the participant’s home. If interviews are to take place at a participant’s home, the researcher will first discuss any immediate or peripheral safety issues with support providers or relevant professionals and clarify if it is safe for them to conduct interviews in the home. Researchers will have access to a mobile phone and screech alarm at all times during interviews and will give details of interview locations, start times and approximate end times to colleagues at their research department. After the interviews, researchers will ask the participants how they feel and if they would like to discuss anything further with their responsible clinician.

### Confidentiality

All participants will be interviewed in private by trained researchers. The information provided by participants will be confidential and anonymised. In some situations, however, it may be necessary to disclose personal information without a patient’s consent if it is in the public interest (i.e. where a failure to do so may expose the patient or others to risk of death or serious harm). The limits of confidentiality are explained on the participant information sheet and will be discussed with all participants as part of the informed consent process. The General Medical Council guidance on confidentiality will be followed [45]; disclosure of personal information without consent may be justified in the public interest where failure to do so may expose the patient or others to risk of death or serious harm. In cases where the patient or others are exposed to a risk so serious that it outweighs the patient’s privacy interest, consent to disclose will be sought. If it is not practicable to obtain consent, the information will be disclosed to an appropriate person or authority. The researchers will contact one of the clinical applicants on the grant to discuss any situations when confidentiality may need to be broken. Detailed guidance on safeguarding maternal and child welfare is outlined in the Programme-wide Standard Operating Procedures.

### Trial status

The trial is currently recruiting.

## Appendix

### Psychological Wellbeing Practitioner perinatal competencies for the DAWN trial

A pre-requisite for working on the trial was that the Psychological Wellbeing Practitioners (PWPs) had completed PWP (IAPT) low intensity training and had acquired a Postgraduate Certificate in Low Intensity Cognitive Behavioural Interventions according to the National Curriculum for the Education of Psychological Wellbeing Practitioners (now the 3^rd^ edition, 2015). On appointment to the trial, the PWPs received training to cover competencies relevant to working with women during the perinatal period, as specified below.

### Knowledge of pregnancy, childbirth and the postnatal period

This competency includes knowledge of:

- Physical and emotional changes during the perinatal period
- Common complications of pregnancy, childbirth and the postnatal period
- Routine maternity and obstetric care

### Knowledge of mental health during the perinatal period

This competency includes knowledge of:

- Prevalence of mental health problems; risk of relapse; the impact of mental health problems on the infant
- Understanding of the importance of prevention and early intervention and the role of specialist perinatal mental health services
- Knowledge of depression and anxiety disorders and how they may present in the perinatal period as well as how their course can fluctuate in response to events in the perinatal period
- Knowledge of serious mental health problems and how they may present in the perinatal period including bipolar disorder and post-partum psychosis.

### Understanding of the parent-infant relationship

This competency involves awareness of issues relating to infant mental health, attachment and parenting (including the ways in which experiences of being parented are relevant when becoming a parent).

### Therapeutic competencies relating to the perinatal period

These competencies include:

- The ability to support women using Guided Self Help materials with adaptations for the perinatal period
- Knowledge of behavioural activation that is appropriate for the woman’s goals, her stage of pregnancy or for the postnatal period allowing for taking care of the baby
- Knowledge of relaxation techniques
- Knowledge of Guided Self Help interventions to increase the availability of interpersonal support
- The ability to work with cognitions and beliefs about the baby/about self as a parent
- Knowledge of Guided Self Help interventions relating to health and lifestyle.

### Metacompetencies

Metacompetencies include planning and delivering treatment in a flexible manner, including adjusting to unanticipated events during pregnancy or postnatally.

### Professional, ethical and legal issues

This competency includes:

- Knowledge of IAPT including criteria for stepping up and down relevant to the perinatal period
- Knowledge of local specialist perinatal services, referral routes and care pathways
- Awareness of national and local organisations relevant to the perinatal period
- Ability to liaise with other agencies such as maternity services, health visitors and social care
- Knowledge of safeguarding, including assessing and managing risks to the unborn infant and making referrals to Social Care
- Ability to administer, score and interpret routine outcome measures
- Awareness of issues relating to consent and confidentiality
- Awareness of equality and diversity, including cultural differences in parenting, same sex couples and single parents.

### DAWN trial induction and specialist training

This training includes:

- Overview of perinatal mental health
- Adaptations of CBT and common themes in the perinatal period
- Use of the workbook
- Supporting Infant Mental Health
- Safeguarding Children Level 3
- Domestic Abuse Awareness
- Smoking Cessation
- Smoking Cessation in Pregnancy and the Postpartum Period
- Observing a booking appointment
- Visit to the antenatal and postnatal wards.

## Additional files

Additional file 1: Table S1. SPIRIT checklist. (DOC 121 kb)
Additional file 2: Figure S1. SPIRIT figure: schedule of enrolment, intervention and assessments. (DOCX 23 kb)
Additional file 3: Figure S2. Protocol flow diagram. (PPTX 65 kb)
Additional file 4: Table S2. List of study measures. (DOCX 45 kb)

## Abbreviations

CBT: Cognitive behavioural therapy
DMEC: Data Monitoring and Ethics Committee
EPDS: Edinburgh Postnatal Depression Scale
GAD-7: Generalised Anxiety Disorder scale
IAPT: Improving Access to Psychological Therapies
NICE: National Institute for Health and Care Excellence
PHQ-9: Patient Health Questionnaire
PWP: Psychological Wellbeing Practitioner
REC: Research Ethics Committee
SCID: Structured Clinical Interview DSM-IV
