# Supplementary material for: Depression: an exploratory parallel-group randomised controlled trial of Antenatal guided self help for WomeN (DAWN): study protocol for a randomised controlled trial
Source: Trials. 2016 Oct 18;17:503. doi: 10.1186/s13063-016-1632-6 (PMC5070149; doi:10.1186/s13063-016-1632-6)
Supplement: Additional file 2: Figure S1. — SPIRIT figure: schedule of enrolment, intervention and assessments. (DOCX 23 kb) [file 13063_2016_1632_MOESM2_ESM.docx]

|  | Enrolment | Allocation | Post-allocation | |
| --- | --- | --- | --- | --- |
| TIMEPOINT | -t_1_ | 0 | 14 weeks post randomisation | 3 months post-delivery |
| ENROLMENT: |  |  |  |  |
| Eligibility screen | X |  |  |  |
| Informed consent | X |  |  |  |
| Baseline questionnaire | X |  |  |  |
| Randomisation and allocation |  | X |  |  |
| INTERVENTIONS: |  |  |  |  |
| Guided Self Help (plus treatment as usual) |  |  |  |  |
| Treatment as usual |  |  |  |  |
| ASSESSMENTS: |  |  |  |  |
| Baseline measures | X |  |  |  |
| Primary outcomes |  |  | X |  |
| Secondary outcomes |  |  | X | X |

**Additional file 2: Figure S1: SPIRIT Figure - schedule of enrolment, intervention and assessments**
