# Supplementary material for: Depression: an exploratory parallel-group randomised controlled trial of Antenatal guided self help for WomeN (DAWN): study protocol for a randomised controlled trial
Source: Trials. 2016 Oct 18;17:503. doi: 10.1186/s13063-016-1632-6 (PMC5070149; doi:10.1186/s13063-016-1632-6)
Supplement: Additional file 3: Figure S2. — Protocol flow diagram. (PPTX 65 kb) [file 13063_2016_1632_MOESM3_ESM.pptx]

## Slide 1
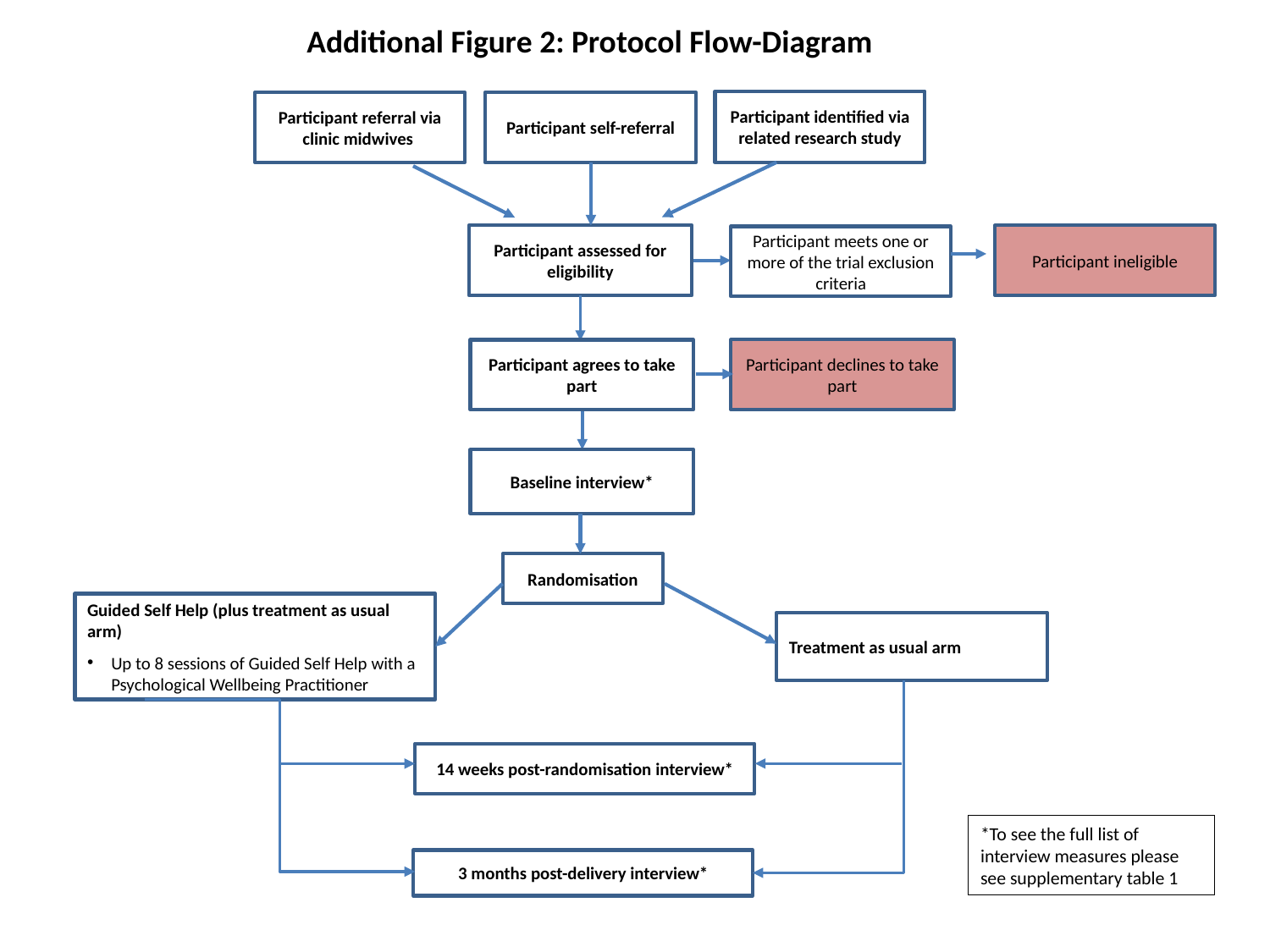

Additional Figure 2: Protocol Flow-Diagram
Participant identified via related research study
Participant referral via clinic midwives
Participant self-referral
Participant assessed for eligibility
Participant ineligible
Participant meets one or more of the trial exclusion criteria
Participant declines to take part
Participant agrees to take part
Baseline interview*
Randomisation
Guided Self Help (plus treatment as usual arm)
Up to 8 sessions of Guided Self Help with a Psychological Wellbeing Practitioner
Treatment as usual arm
14 weeks post-randomisation interview*
*To see the full list of interview measures please see supplementary table 1
3 months post-delivery interview*
