# Supplementary material for: Depression: an exploratory parallel-group randomised controlled trial of Antenatal guided self help for WomeN (DAWN): study protocol for a randomised controlled trial
Source: Trials. 2016 Oct 18;17:503. doi: 10.1186/s13063-016-1632-6 (PMC5070149; doi:10.1186/s13063-016-1632-6)
Supplement: Additional file 4: Table S2. — List of study measures. (DOCX 45 kb) [file 13063_2016_1632_MOESM4_ESM.docx]

**Additional file 4: Table S2: List of Study Measures**

| **Name of Instrument** | **Time of Administration** | | | **Instrument Details** |
| --- | --- | --- | --- | --- |
|  | **Baseline** | **14 weeks** | **3 months** |  |
| Socio-demographic characteristics (including smoking behaviours)* | **X** |  |  | a researcher-administered questionnaire, developed by the study team to collect information on key socio-demographics, obstetric data and pregnancy risk factors (including smoking behaviours) |
| Smoking behaviours |  | **X** | **X** | A four-item self-reported questionnaire, developed by the study team, to collect information on past and current smoking behaviours |
| EPDS* | **X** | **X** | **X** | a ten-item self-administered screen for perinatal depression, validated in 20 languages ([28](#_ENREF_28)). The EPDS has a positive predictive value for postnatal major depression of 9-64% (cut-off score 9/10) or 17-100% (cut-off score 12/13) and for antenatal major depression 60-80% (cut-off score 14/15) ([29](#_ENREF_29)) |
| Whooley | **X** | **X** | **X** | a self-administered questions comprise two items that measure symptoms of depression: (1) “during the past month, have you often been bothered by feeling down, depressed or hopeless?”, and (2) “during the past month, have you often been bothered by little interest or pleasure in doing things?” ([26](#_ENREF_26)) The Whooley questions have demonstrated good sensitivity and specificity (0.97 and 0.95, respectively) with the Composite International Diagnostic Interview (CIDI) ([30](#_ENREF_30)), and the addition of a “help” question (i.e. “is this something you feel you need or want help with?”) has shown to improve specificity ([31](#_ENREF_31)) |
| SCID-I for DSM-IV Axis I Disorders* | **X** |  |  | a semi-structured diagnostic interview widely used in psychiatric research ([33](#_ENREF_33), [34](#_ENREF_34)), including national epidemiological studies ([35](#_ENREF_35), [36](#_ENREF_36)). The interview consists of standardised diagnostic questions arranged in modules corresponding to each DSM-IV Axis I disorder ([37](#_ENREF_37)). The test-retest reliability of SCID-I generally falls between reported values for similar instruments such as the Diagnostic Interview Schedule. This study will use only the Mood Episodes, Mood Disorders and Anxiety Disorders modules; diagnostic clusters for the following disorders will be used: (1) current major and minor depressive disorders, (2) bipolar I and II disorder, (3) panic disorder, (4) agoraphobia, social phobia, specific phobia, (5) obsessive compulsive disorder, (6) post-traumatic stress disorder and current acute stress disorder, (7) generalised anxiety disorder and mixed anxiety depressive disorder([32](#_ENREF_32)); |
| SCID-I for DSM-IV Eating Disorders module* | **X** |  |  | a researcher-administered standardised assessment for making accurate diagnoses of DSM-IV Axis I eating disorders, including anorexia nervosa, bulimia nervosa and binge eating disorder. The SCID-I Eating Disorders module will be used to assess the sensitivity, specificity and positive predictive value of the adapted SCOFF questionnaire in identifying eating disorders in the antenatal period; |
| SCID-I for DSM-IV Borderline Personality Disorders* | **X** |  |  | a researcher-administered standardised assessment for making accurate diagnoses of DSM-IV Axis II borderline personality disorders. The SCID-II Borderline Personality Disorders sub-section contains nine-items and will be used to estimate the prevalence of borderline personality disorder at antenatal booking; |
| adapted version of the SCOFF questionnaire* | **X** |  |  | adapted for use with antenatal populations ([39](#_ENREF_39)), is an assessment tool which identifies attitudes and behaviours associated with possible eating disorders. The tool comprises six items: (1) “do you make yourself sick, use laxatives or other medication to avoid weight gain or for weight loss?” (2) “do you worry you have lost control over how much you eat?” (3) “are you worried about putting weight on?” (4) “has anyone been worried about your weight being low recently?” (5) “do you believe yourself to be fat when others say you are too thin?” (6) “would you say that food dominates your life?”  Each item is scored either one = “yes” or zero = “no”, and a score of two or above is indicative of a probable eating disorder ([38](#_ENREF_38)). The tool takes approximately 30 seconds to complete ([40](#_ENREF_40)); |
| Standardised Assessment of Personality– Abbreviated Scale* | **X** |  |  | an eight-item researcher-administered screening questionnaire which provides a validated measure of personality dysfunction ([41](#_ENREF_41)). The eight items correspond to a descriptive statement about the person and are scored either 0 = “no” or 1 = “yes”; the eight items are added together to produce a total score of between 0 and 8. A score of 3 or more on the SAPAS indicates probable personality disorder ([41](#_ENREF_41)). The measure demonstrated good sensitivity and specificity (0.94 and 0.85, respectively) with the SCID ([41](#_ENREF_41)); |
| Posttraumatic Stress Disorder Scale* | **X** | **X** | **X** | a 49-item self-administered questionnaire measuring lifetime exposure to traumatic events, as well as providing a diagnosis of PTSD and posttraumatic stress symptom severity in the past month ([42](#_ENREF_42)). The measure is separated into four parts - part one contains 13 items and is completed by all respondents and parts two to four (containing a total of 36 items) are only completed by those reporting exposure to a traumatic event. The PDS has six components: (1) PTSD diagnosis, (2) symptom severity score, (3) number of symptoms endorsed, (4) specifiers related to onset and duration of symptoms, (5) symptom severity rating and (6) level of impairment in functioning; criterion must be met for each of the six components. The PDS demonstrated strong internal consistency (Cronbach’s α ≥ 0.78) and good sensitivity and specificity (0.89 and 0.75, respectively) with the SCID ([42](#_ENREF_42), [43](#_ENREF_43)). This measure has previously been used in prenatal populations ([44](#_ENREF_44)); |
| Alcohol Use Disorders Identification Test* | **X** |  |  | a ten-item self-administered questionnaire of alcohol-related use in the previous year ([35](#_ENREF_35)). The 10 items include two questions on quantity and frequency of alcohol use, a question on binge drinking, two CAGE questions, and five DSM-III criterion questions. The AUDIT is rated on a five-point scale; the first item is scored from 0= “never” to 4 = “4 or more times a week”; the second item from 0 = “1 to 2” to 4 = “ten or more” and the following six items from 0= “never” to 4 “daily or almost daily”. The final two items are rated on a three-point scale: 0= “no”, 2=”yes, but not in the past year”, and 4= “yes during the past year” ([35](#_ENREF_35)). The maximum score for the AUDIT is 40, with a score of 6 or more for women, and 8 or more for men, indicating harmful or hazardous drinking. A score of 13 or more in women, and 15 or more in men, is likely to indicate alcohol dependence ([35](#_ENREF_35)). Using a cut-off score of 8, the measure demonstrated good sensitivity and specificity (0.85 and 0.88, respectively) with ICD-10 diagnostic criteria for harmful drinking ([36](#_ENREF_36)). |
| Alcohol Use Disorders Identification Test-C |  | **X** | **X** | a modified version of the AUDIT measure and contains three self-administered questions on alcohol-related use in the previous year ([45](#_ENREF_45)). The three items include one question on the frequency of alcohol use and two questions on the quantity of alcohol use. The AUDIT is rated on a five-point scale; the first item is scored from 0= “never” to 4 = “4 or more times a week”; the second item from 0 = “1 to 2” to 4 = “ten or more” and the third item from 0= “never” to 4 “daily or almost daily” ([45](#_ENREF_45)). The AUDIT-C is scored on a scale from 0-12; a score of three or more for women is indicative of hazardous drinking; |
| Drug Use Disorders Identification Test* | **X** |  |  | an 11-item self-administered questionnaire of drug-related problems in the previous year ([46](#_ENREF_46)). The DUDIT is rated on a five-point scale; the first two items are scored from 0= “never” to 4 = “4 or more times a week”; the third item from 0 = “0” to 4 = “seven or more”, and the following six items from 0= “never” to 4 “daily or almost every day”. The final two items are rated on a three-point scale: 0= “no”, 2=”yes, but not over the past year”, and 4= “yes over the past year” ([46](#_ENREF_46)). The maximum score for the DUDIT is 44, with a score of 2 or more for women, and 6 or more for men, indicating drug-related problems. A score of 25 or more in women, for either sex, is likely to indicate drug dependence ([46](#_ENREF_46)). Using a cut-off score of 25, the measure demonstrated good sensitivity and specificity (0.90 and 0.78, respectively) with ICD-10 diagnostic criteria for harmful drinking ([46](#_ENREF_46)); |
| Composite Abuse Scale - short version* | **X** | **X** | **X** | an 11-item self-administered questionnaire of abusive partner behaviours in the year before pregnancy and during pregnancy ([47](#_ENREF_47), [48](#_ENREF_48)). Items are presented in a six-point format requiring respondents to answer 0= “never”, 1= “only once”, 2 = “several times”, 3 = “monthly”, 4 = “weekly” or 5 = “daily” ([47](#_ENREF_47), [48](#_ENREF_48)). Two total scores, ranging from 0-55, are calculated for abuse prior to pregnancy and abuse during pregnancy; total scores are obtained by adding scores for all items ([49](#_ENREF_49)). A cut-off score of three or more is indicative of abuse ([49](#_ENREF_49)); |
| Social Provisions Scale* | **X** | **X** | **X** | a 24 item researcher-administered measure of perceived social support ([50](#_ENREF_50)). The measure covers six components of social support: (1) attachment (receiving a sense of security and safety); (2) social integration (feeling a sense of belonging to a group of individuals with shared interests and concerns); (3) opportunity for nurturance (feeling a need to provide for the well-being of another); (4) reassurance of worth (feeling important to or valued by others); (5) reliable alliance (feeling able to count on others for assistance, if necessary); and (6) guidance (receiving advice and information from trusted individuals). Items are rated on a 4-point likert scale ranging from 1 = “strongly disagree” to 4 = “strongly agree” ([50](#_ENREF_50)). Total scores are calculated by summing all of the responses; scores range from 24–96, with higher scores indicating more perceived social support. This measure has previously been used in antenatal populations ([51](#_ENREF_51), [52](#_ENREF_52)); |
| 36 item Short Form Health Survey* | **X** | **X** | **X** | a questionnaire constructed for self-administration by persons 14 years of age and older, and for administration by a trained inter-viewer in person or by telephone ([53](#_ENREF_53)). It measures health on eight multi-item dimensions: 1) limitations in physical activities because of health problems; 2) limitations in social activities because of physical or emotional problems; 3) limitations in usual role activities because of physical health problems; 4) bodily pain; 5) general mental health (psychological distress and well-being); 6) limitations in usual role activities because of emotional problems; 7) vitality (energy and fatigue); and 8) general health perceptions ([54](#_ENREF_54)). Higher scores indicate better quality of life. The questionnaire has shown to be a valid and efficient measure of health-related quality of life in the maternity context ([55](#_ENREF_55)). The SF-36 will be used to derive SF-6D scores. The SF-6D is a preference-based single index measure of health-related quality of life from which quality adjusted life years (QALYs) can be calculated, through the application of a set of preference weights obtained from a sample of the general population ([56](#_ENREF_56)). |
| EQ-5D-5L* | **X** | **X** | **X** | a preference-based measure of health related quality of life measured on five dimensions (mobility, self-care, usual activities, pain/discomfort and anxiety/depression), each rated on five levels (no problems, slight problems, moderate problems, severe problems and extreme problems) ([57](#_ENREF_57)). Participants are classified into one of 3125 health states. The EQ-5D-5L is considered more sensitive to change than the original three level version (EQ-5D-3L), but it can still be used to derive EQ-5D-3L scores which can be used to calculate quality adjusted life years (QALYs) ([58](#_ENREF_58), [59](#_ENREF_59)). The EQ-5D-3L measure has been extensively used in health economic evaluations and its psychometric properties are adequate ([60](#_ENREF_60)), although it’s validity, and the validity of the EQ-5D-5L in postnatal depression populations is unclear. For this reason, a comparison between the SF-6D and the EQ-5D will be undertaken. General population preference weights are not currently available for the EQ-5D-5L, but these may become available during the course of this study. |
| PHQ-9 | **X** | **X** | **X** | a 9-item self-administered measure of depression which rates the frequency and severity of depressive symptoms ([61](#_ENREF_61)). The nine questions focus on bothersome feelings, thoughts about behaviours over the past two weeks, rated on a four-point scale from 0 = “not at all” to 3 = “nearly every day”. PHQ scores ≥ 10 are found to have a sensitivity of 88% and a specificity of 88% for major depression ([61](#_ENREF_61)); |
| Generalized Anxiety Disorder Scale | **X** | **X** | **X** | a seven-item self-administered screening questionnaire measuring the presence and severity of common anxiety disorders, including generalised anxiety, panic, social anxiety and posttraumatic stress disorder ([62](#_ENREF_62)). The items of the GAD-7 are scored from 0 = ‘not at all’ to 3 = ‘nearly every day’, with scores ranging from 0 to 21. A cut-off score of five represents mild symptoms, ten represents moderate symptoms and 15 represents severe anxiety symptoms ([63](#_ENREF_63)); |
| Adult Service Use Schedule (AD-SUS) |  | **X** | **X** | A modified version of an Adult Service Use Schedule (AD-SUS) was used to collect information on resource use is an economic evaluation questionnaire measuring use of resources (i.e., the number and length of contacts with health and social services) (51). The ADSUS in this study was specifically modified to cover antenatal services as well as general health and social services. Specifically, the ADSUS covered midwifery services pre and post birth, maternity services, plus the following general health and social services care provided for mother and/or baby: accommodation; hospital based services; and community based services. The questionnaire is separated in to four components: (1) accommodation use, (2) medication use, (3) hospital service use, and (4) community service use ([68](#_ENREF_68)); |
| IAPT phobia scale ([69](#_ENREF_69)) | **X** | **X** | **X** | a self-administered measure of severity of phobias routinely used in IAPT. The items of the measure examine the avoidance of situations or objects. Items are rated on a nine-point scale from 0 = “would not avoid it” to 8 = “always avoid it”; total scores are summed and range from 0-24 ([69](#_ENREF_69)); |
| Work and Social Adjustment Scale (WASAS) | **X** | **X** | **X** | a five-item self-administered measure of general impairment ([70](#_ENREF_70)) routinely used in IAPT. The items of the measure examine the impact of depression within the context of work, home management and social and leisure activities. Items are rated on a nine-point scale from 0 = “not at all” to 8 = “very severely”; total scores are summed and range from 0-40; |
| Mother-infant interactions |  |  | **X** | captured in a 5-10 minute video clip taken during play at home and subsequently assessed by a trained rater using the CARE Index. Coding of the interaction takes between 15-20 minutes and assesses three mother scales (sensitivity, control and unresponsiveness) and four infant scales (cooperativeness, compulsivity, difficultness and passivity) ([71](#_ENREF_71)); |
| Postpartum Bonding Questionnaire (PBQ) |  |  | **X** | a 25 item self-administered measure designed to provide an early indication of difficulties within mother-infant relationships, through the assessment of a mother’s feelings and attitudes towards her infant ([64](#_ENREF_64)). Items are rated on a six-point scale from 0 = “always” to 5 = “never”; when the statement reflects a negative emotion or attitude, the scoring is reversed. The 25 items comprise four sub-scales: (1) impaired bonding (12 items, scores ranging from 0 to 60), (2) rejection and anger (7 items, scores ranging from 0 to 35), (3) anxiety about care (4 items, scores ranging from 0 to 20), and (4) risk of abuse (2 items, scores ranging from 0 to 10). Total scores are calculated by summing the 25 items (scores range from 0 to 125); sub-scale score cut-offs are set at 17 for ‘impaired bonding’, two for ‘rejection and anger’, ten for ‘anxiety about care’ and three for ‘risk of abuse’ ([64](#_ENREF_64)). Analysis of the scale includes both total and subscale scores. This measure has been translated in to several languages and is frequently used in research on mother-infant bonding with postpartum populations ([65](#_ENREF_65), [66](#_ENREF_66)) and its psychometric properties evaluated ([67](#_ENREF_67)); |
| Pregnancy-Related Thoughts | **X** | **X** |  | a ten-item self-administered questionnaire which assesses the frequency with which women worry about their health, their baby’s health, labour and delivery and caring for the baby. Items are rated on a four-point scale from 1 = “never/not at all” to 4 = “a lot of the time/very much”. Total scores are calculated by summing the ten items (ranging from 10 to 40), with higher scores indicating higher anxiety. This measure has acceptable internal consistency (Cronbach α = 0.78) ([72](#_ENREF_72)); |
| Prenatal Attachment Inventory | **X** | **X** |  | a 21-item self-administered questionnaire which assesses a woman’s thoughts, feelings and relationship to their foetus. Items are rated on a four-point scale from 1 = “almost never” to 4 = “almost always”. Total scores are calculated by summing the 21 items (ranging from 21 to 84), with higher scores indicating greater attachment ([73](#_ENREF_73)); |
| Metacognitive awareness | **X** | **X** | **X** | a nine-item self-administered questionnaire measuring the extent to which a person recognises that their negative depressive thoughts and feelings might not reflect actual realities. Items are rated on a seven-point Likert scale from 1 = “totally agree” to 7 = “totally disagree”. Total scores are calculated by summing the nine items (ranging from 9 to 63), with higher scores reflecting greater metacognitive awareness ([74](#_ENREF_74)); |

*This denotes the additional baseline questionnaires that are administered to participants that are referred via clinical midwives or via self-referral. Participants that are taking part in a related study will have already completed the questionnaires as part of their assessment for eligibility for the trial.
